# Integrating an Interventional Pain Management Curriculum in Hospice and Palliative Medicine Fellowship Training: A Feasibility Study

**DOI:** 10.1177/10499091241268597

**Published:** 2024-07-29

**Authors:** Emily Marquez Campbell, Chaitanya Konda, Kelsey Lau, Winnie Wang

**Affiliations:** 1 Department of Physical Medicine and Rehabilitation, 12334 University of Texas at Southwestern Medical Center, Dallas, TX, USA; 2 Department of Internal Medicine, Palliative Care Division, 12334 University of Texas at Southwestern Medical Center, Dallas, TX, USA

**Keywords:** cancer pain, interventional pain management, palliative medicine, graduate medical education

## Abstract

**Background:**

Pain is a common symptom for patients with cancer. Hospice and Palliative Medicine (HPM) physicians are expected to be experts in both pharmacologic and non-pharmacologic treatment of pain for this patient population. Insufficient knowledge of non-pharmacologic, interventional approaches to pain management is a barrier to providing optimal care. This study assesses the feasibility and effectiveness of an interventional pain management curriculum on HPM fellow knowledge at a single institution.

**Objectives:**

The primary objective was to implement an interventional pain management curriculum for HPM fellows’ and secondly to measure its effects on their knowledge and confidence in interventional pain management approaches.

**Methods:**

We executed an interventional pain management curriculum for HPM fellows. The curriculum consisted of 6 fifty-minute virtual lectures. Anonymous pre- and post-curriculum surveys were used to assess curricular impact.

**Results:**

Post-course surveys showed a significant increase in HPM fellows’ knowledge and confidence in interventional pain management techniques.

**Conclusions:**

An interventional pain management curriculum for HPM fellows is a feasible and promising intervention to significantly impact fellows’ knowledge and confidence in non-pharmacologic treatment of cancer pain.

## Introduction

Pain is a prevalent symptom in patients with cancer that has a profound impact on patients’ quality of life and function.^1,2^ The task of treating pain in patients with advanced cancer is complex and requires a multimodal approach, especially for pain that is refractory to pharmacologic management.^3-6^ In acknowledgment of this, the World Health Organization recently updated its analgesic ladder framework for treatment of cancer-related pain to include a fourth step: invasive and minimally invasive treatments, which include numerous non-pharmacologic procedures for treating persistent pain.^7^ Hospice and Palliative Medicine (HPM) specialists receive training in expert management of pain in patients with advanced cancer and other serious illnesses. Knowledge of indications, toxicities and appropriate referral for interventional pain management procedures is listed as a core competency for HPM fellowship training programs.^8^ Despite this, it has been shown that the use of interventional pain procedures in the management of complex cancer-related pain is underutilized.^9^ In national surveys, the barriers to regular integration of interventional pain management procedures in the management plans of cancer-related pain are variable but include lack of experience of HPM specialists.^10,11^ It has been proposed that further education and training in aspects of interventional therapies is needed.^12^ However, to our knowledge, there have been no published studies to inform the approach for incorporating interventional pain management education into HPM fellowship training effectively. In this single pilot study, we set out to determine the feasibility of implementing a brief interventional pain management curriculum and to assess its effects on HPM fellows’ confidence in their knowledge of non-pharmacologic procedures for management of cancer-related pain. Similar interventions with a focus on palliative radiotherapy education have been shown to be feasible and effective.^13^

## Methods

### Setting and Course Design

We sought to develop and implement an interventional pain management curriculum for HPM fellows. The study was formally submitted to our center’s Institutional Review Board (IRB) and determined to meet exempt criteria under 45 CFR 46.104(d). We developed and implemented a virtual interventional pain management curriculum for HPM fellows at our institution. The course consisted of 6 fifty-minute virtual lectures delivered by an expert group of physician faculty, all board-certified in either Physical Medicine and Rehabilitation (PM&R) or Anesthesiology, with a subspecialty certification in Pain Medicine. The content of the 6 lectures was selected in a collaborative fashion by involved faculty to address aspects of interventional pain management most relevant to HPM physicians, including: regional anesthesia procedures, central ganglion denervation procedures, basic spinal procedures, intrathecal therapies, neuromodulation, and vertebroplasty and kyphoplasty. Throughout all lectures, the background of interventions, indications, contraindications, and techniques were discussed in depth and augmented further with case-based discussion. Afterwards, HPM fellows had the opportunity to ask questions of the faculty presenters. The virtual lectures were also recorded, and lecture slides were provided to fellows for future reference. (Table 1)

**Table 1.** Interventional Pain Management Lecture Topics.

Basic Spine Procedures
Intrathecal Therapies
Neuromodulation Spinal Cord Stimulation/Peripheral Nerve Stimulation
Regional Pain Procedures
Sympathetic Blocks with Neurolysis/Radiofrequency ablation
Vertebroplasty and Kyphoplasty

.

### Participants

Two cohorts of HPM fellows, comprised of 4 fellows total for the first cohort during the first year of the study and 6 fellows total for the second cohort during the second year of the study, completed the pre-course survey, the curriculum in its entirety, and the post-course survey. Nine of the HPM fellows participated in the virtual curriculum in real-time. One HPM fellow was unable to participate in real-time due to parental leave, but they were able complete the virtual curriculum asynchronously by watching the lecture video recordings and reviewing lecture slides.

### Measurements

Each fellow completed a six-item, anonymous pre-course electronic survey, completed within 1 week prior to the first lecture. Each fellow also completed the same survey post-course within 1 week after the last lecture. The questions utilized a 5-point Likert scale to assess confidence levels (1 = Strongly disagree; 5 = Strongly agree.) All responses were collected in a de-identified fashion into a secure REDCap database. The Wilcoxon rank-sum test was then utilized to compare pre- and post-test confidence levels. A *P* value of <0.05 was used to determine statistical significance. We hypothesized that after the course, fellows would report significantly more confidence in their knowledge of interventional pain management interventions.

## Results

Prior to the course, all fellows either agreed or strongly agreed that interventional pain management specialists are important as collaborators with the Palliative Care team. Despite this, prior to the course, the majority of the fellows disagreed regarding their confidence in their knowledge of when to refer patients to an interventional pain management specialist or to identify which patients would benefit from a referral. The majority of the fellows also reported they disagreed regarding their confidence in their knowledge of the indications and options for interventional pain management procedures and in their ability to discuss these options with specialists.

**Figure 1.**
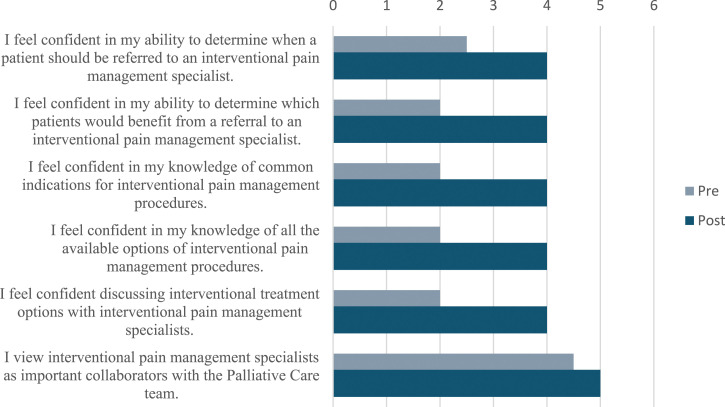
Results from the pre- and post-curriculum surveys, in which participants’ responses were recorded via Likert scale (1 = Strongly disagree, 2 = Disagree, 3 = Neutral, 4 = Agree, 5 = Strongly agree) reported as median scores.

Post-course surveys demonstrated a significant increase in HPM fellow confidence levels in all areas queried. Thefellows reported that they agreed that they were confident in their knowledge of when patients should be referred for interventional pain management procedures (*P* = 0.007) and in which patients would benefit (*P* = 0.007). They also all agreed that they were confident in their knowledge of indications for (*P* = 0.008) and options of interventional pain management procedures (*P* = 0.013), and in their ability to discuss these with pain specialists (*P* = 0.008). (Figure 1)

.

## Discussion

This single-institution feasibility study demonstrated the ability of a simple curriculum intervention to significantly increase the confidence and knowledge of HPM fellows in non-pharmacologic pain management techniques. Specifically, there was improvement in the areas of knowledge of timing and indications for interventional pain management referrals, appropriate selection of patients who may benefit, and in knowledge of treatment options and ability to navigate discussions with specialists. There was no change demonstrated in the fellows’ acknowledgement that interventional pain specialists are important collaborators with the interdisciplinary Palliative Care team, which was agreed upon or strongly agreed upon even prior to our curriculum intervention.

The American Academy of Hospice and Palliative Medicine created an expert workgroup in 2016 to develop a comprehensive list of twenty-two curricular milestones to address the call for competency-based approach to graduate medical education. Managing pain and non-pain symptoms using nonpharmacologic strategies, including interventional pain procedures, is considered a key educational component of a comprehensive HPM fellowship training program to prepare fellows to address patient suffering and distress.^14^ To our knowledge, this is the first study of the impact of a curricular intervention designed to address this milestone in an HPM fellowship training program. There is a systematic review of training in symptom management in non-palliative programs that showed pharmacologic strategies for pain management were most frequently covered, not non-pharmacologic strategies.^15^ Of course, it remains a possibility that other HPM fellowship programs are providing similar interventional pain management curriculum and have not yet reported on their experience. In a similar feasibility study of a curricular intervention on HPM fellow’s knowledge of palliative radiotherapy, subsequent studies were performed including a national needs assessment via a cross-sectional survey of HPM fellows on palliative radiotherapy education^16^ and a cross-sectional national survey of HPM fellowship program directors to further characterize palliative radiotherapy education needs and offerings.^17^ Conducting similar studies in regards to interventional pain management education could provide further valuable insight and knowledge that builds on our study findings.

Strengths of our study include ease of implementation of intervention and the collaborative, interdisciplinary approach to curriculum development. Extra effort was required to coordinate with interventional pain specialists during curricular development and execution, but their expertise proved to enrich the curriculum immensely. We also showed that positive results can be achieved with a relatively low number of individual lectures, which is important given the typical length of HPM fellowship (1 year) imposes somewhat of a time constraint. Limitations include small survey size, given it was performed only at 1 institution and lack of a longitudinal assessment to demonstrate the longevity of positive impacts of curriculum intervention. However, we did attempt to address the small survey size and improve the generalizability of our results by performing the intervention with 2 cohorts of fellows and pooling the data. Despite this, due to small sample size overall, though best efforts were made to protect anonymity, an element of bias could exist in that fellows might be less likely to report negative responses to their colleagues and mentors who created and implemented the curriculum.

Given the subjective nature of pain and the complex physiologic factors contributing to the clinical symptoms, it is essential to be familiar with a variety of modalities to manage cancer-related pain. As clinicians continue to face the challenges of providing pain relief in the setting of disease progression in patients with cancer, targeting the physiologic responses while also integrating spiritual and psychological components at play,^18^ the importance of understanding the appropriate setting for interventional pain management procedures cannot be overstated. As Bhatnagar and Gupta reported, “Interventional therapies have a specific role in management of cancer pain…rather than considering it as a standalone therapeutic measure, it should be considered as an indispensable component of multimodal pain management strategy.”^19^ Assuring that HPM providers are able to assess and determine how to best meet the needs of patients with cancer-related pain is an essential part of fellowship training. Providing the tools and resources to ensure that the pain is adequately assessed and treated will not only potentially benefit the patient’s physical health, it can also impact their quality of life.

## Conclusion

An interventional pain management curriculum in a Hospice and Palliative Medicine program is feasible to implement in HPM fellowship programs and significantly improved fellows’ knowledge and confidence in non-pharmacologic pain management approaches. Future directions could include implementing the curriculum at multiple sites, expanding participant pool to include other members of the interdisciplinary Palliative Care team, the creation of longitudinal assessment, and a national interventional pain education in HPM fellowship program survey and needs assessment.
